# Supplementary material for: Development and application of decontamination methods for the re-use of laboratory grade plastic pipette tips
Source: PLoS One. 2024 Dec 31;19(12):e0301950. doi: 10.1371/journal.pone.0301950 (PMC11687887; doi:10.1371/journal.pone.0301950)
Supplement: Attachment — Submitted filename: Response to Reviewers Pipette.docx [file pone.0301950.s002.docx]

Original Email:

PONE-D-24-08899

Development and Application of Decontamination Methods for the Re-Use of Laboratory Grade Plastic Pipette Tips

PLOS ONE
Dear Dr. Lee,
Thank you for submitting your manuscript to PLOS ONE. After careful consideration, we feel that it has merit but does not fully meet PLOS ONE’s publication criteria as it currently stands. Therefore, we invite you to submit a revised version of the manuscript that addresses the points raised during the review process.

**Please address the issues raised by the honorable reviewer.** 

Please submit your revised manuscript by Jul 14 2024 11:59PM. If you will need more time than this to complete your revisions, please reply to this message or contact the journal office at <a href="mailto:plosone@plos.org">plosone@plos.org. When you're ready to submit your revision, log on to https://www.editorialmanager.com/pone/ and select the 'Submissions Needing Revision' folder to locate your manuscript file.

Please include the following items when submitting your revised manuscript:

- A rebuttal letter that responds to each point raised by the academic editor and reviewer(s). You should upload this letter as a separate file labeled 'Response to Reviewers'.
- A marked-up copy of your manuscript that highlights changes made to the original version. You should upload this as a separate file labeled 'Revised Manuscript with Track Changes'.
- An unmarked version of your revised paper without tracked changes. You should upload this as a separate file labeled 'Manuscript'.

If you would like to make changes to your financial disclosure, please include your updated statement in your cover letter. Guidelines for resubmitting your figure files are available below the reviewer comments at the end of this letter.

If applicable, we recommend that you deposit your laboratory protocols in protocols.io to enhance the reproducibility of your results. Protocols.io assigns your protocol its own identifier (DOI) so that it can be cited independently in the future. For instructions see: <https://journals.plos.org/plosone/s/submission-guidelines#loc-laboratory-protocols>. Additionally, PLOS ONE offers an option for publishing peer-reviewed Lab Protocol articles, which describe protocols hosted on protocols.io. Read more information on sharing protocols at <https://plos.org/protocols?utm_medium=editorial-email&utm_source=authorletters&utm_campaign=protocols>.

We look forward to receiving your revised manuscript.

Kind regards,

Abdul Rauf Shakoori
Academic Editor
PLOS ONE

Journal Requirements:

1. When submitting your revision, we need you to address these additional requirements.

Please ensure that your manuscript meets PLOS ONE's style requirements, including those for file naming. The PLOS ONE style templates can be found at 
https://journals.plos.org/plosone/s/file?id=wjVg/PLOSOne_formatting_sample_main_body.pdf and 
https://journals.plos.org/plosone/s/file?id=ba62/PLOSOne_formatting_sample_title_authors_affiliations.pdf

2. Thank you for stating the following financial disclosure: 
We would like to thank the ORISE fellowship program for supporting scholars who contributed to this study and FDA Office of the Chief Scientists (OCS)/Medical Counter Measures (MCMi)/OCET and FDA/OST for their funding and support of this study. The funders had no role in study design, data collection and analysis, decision to publish, or preparation of the manuscript.

Please state what role the funders took in the study.  If the funders had no role, please state: "The funders had no role in study design, data collection and analysis, decision to publish, or preparation of the manuscript." 
If this statement is not correct you must amend it as needed. 
Please include this amended Role of Funder statement in your cover letter; we will change the online submission form on your behalf.

[Note: HTML markup is below. Please do not edit.]

Reviewers' comments:

Reviewer's Responses to Questions

**Comments to the Author**

1. Is the manuscript technically sound, and do the data support the conclusions?

The manuscript must describe a technically sound piece of scientific research with data that supports the conclusions. Experiments must have been conducted rigorously, with appropriate controls, replication, and sample sizes. The conclusions must be drawn appropriately based on the data presented.

Reviewer #1: Partly

Reviewer #2: Yes

2. Has the statistical analysis been performed appropriately and rigorously?

Reviewer #1: N/A

Reviewer #2: Yes

3. Have the authors made all data underlying the findings in their manuscript fully available?

The [PLOS Data policy](http://www.plosone.org/static/policies.action#sharing) requires authors to make all data underlying the findings described in their manuscript fully available without restriction, with rare exception (please refer to the Data Availability Statement in the manuscript PDF file). The data should be provided as part of the manuscript or its supporting information, or deposited to a public repository. For example, in addition to summary statistics, the data points behind means, medians and variance measures should be available. If there are restrictions on publicly sharing data—e.g. participant privacy or use of data from a third party—those must be specified.

Reviewer #1: Yes

Reviewer #2: No

4. Is the manuscript presented in an intelligible fashion and written in standard English?

PLOS ONE does not copyedit accepted manuscripts, so the language in submitted articles must be clear, correct, and unambiguous. Any typographical or grammatical errors should be corrected at revision, so please note any specific errors here.

Reviewer #1: Yes

Reviewer #2: Yes

5. Review Comments to the Author

Please use the space provided to explain your answers to the questions above. You may also include additional comments for the author, including concerns about dual publication, research ethics, or publication ethics. (Please upload your review as an attachment if it exceeds 20,000 characters)

**Reviewer #1:** General Comment.

The comparison between traditional methods of cleaning, like detergents, and more recent ones, like UV or CAP, is a subject of outmost interest and value in public health. For instance, CAP have been extensively researched in the last 20 years for different applications, but its actual implementation has evolved more slowly, precisely due to the requirement of more studies about its performance.
The paper has strong points like the organized structure of the manuscript, the thorough description of the protocol and elements used in the biological experiments, as well as the presentation of the results (graphs and tables).
My main concern with the manuscript is that I do not think the authors did a good job in comparing the performance of the three methods. There are parameters that are not consistent, like the number of samples, or seem to be chosen arbitrarily. In addition, there is the perception of lack of knowledge in the explanation of the results that were obtained. Although this is not a physics paper, the authors should have a minimum basic knowledge of the methods they are comparing.
Finally, the paper would benefit from a review by a native speaker who could improve the text and make it more natural and fluid.


Specific comments.

Line 45 and 48: “the highest turnover ratio (95.9 %) ... the second highest turnover ratio (98.4 %)”. There is an incoherence in that statement.

Line 48: 14400 PPM * minutes, remove the ‘s’.

Lines 50 to 52: “Relatively, lower turnover ratio and log reduction of CAP could be attributed to development/optimization of treatment conditions, including increases in exposure time and relative to tip positioning.” It is very difficult to make sense of this sentence, please rewrite.

Line 73: instead of using “[8]; Alconox® is a widely used laboratory cleaner” I would suggest using “[8], which is a widely used laboratory cleaner.” to make the reading more fluid.

Lines 75 and 76: The authors wrote “ozone is a reactive allotrope of oxygen with a lone unpaired electron (free radical) that dimerize with valence electrons on other molecules.” That statement is not correct, please check.

Lines 84 and 85: The authors describe CAP as “a thermal non-equilibrium state between heavy positive ions and electrons achieved [10, 11] through rapid atmospheric pressure discharge.” The statement is not entirely accurate. The presence of negative ions in plasma is very important for Electronegative gases like oxygen and therefore, air. In the method presented in the manuscript, plasma is ignited first using helium gas. However, the decontamination effect is achieved through reactive species of oxygen and nitrogen formed through ionization of the surrounding air.
In addition, they should explain briefly and in a very simple (basic) form how the CAP machine works, or its principle of operation (DBD). In lines 243 and 244 they mention the voltage and frequency used, but without said explanation, that information is entirely useless for the reader.

Line 87: “CAP maintains temperatures of 25- 45°C…”. Please, add references.

Lines 94 to 96: The paragraph should be rewritten, because it is not easy to understand.

Lines 245 to 247: The authors mentioned that the pipette tips were placed 2 to 5 cm under the plasma jet for 1 minute. How and why such distance and exposure time were selected.

Line 368 (table 4): Why are there inconsistencies in the number of samples used (73 vs 63)? The turnover ratio is better for O3 14400 ppm than for detergent, but the authors claim otherwise.
I do not think the authors are making a fair comparison or assessment of the cleaning methods proposed, since they seem to have chosen arbitrary exposure distance and time for CAP. They soaked the tip racks for 30 minutes in the detergent solution and used the same time exposure for O3 14400 ppm; however, for CAP they exposed the pipettes for 1 minute, without further explanation to justify that decision. Furthermore, as mentioned before, CAP decontamination is mostly achieved through the action of ROS/RNS (O3 being among them). UV radiation may have some effect if the object is placed near enough to the plasma luminescence. It would have been very useful if the authors could have roughly measured the concentration of at least some of the ROS/RNS, although I understand that this is only possible if they have access to some specialized equipment. However, the authors at least should have done a preliminary study to establish a minimum exposure time, after which the variations in log reduction are not considerable.

**Reviewer #2:** The experiment and data presented is good to be accepted as it is. The experiment and statistical analysis were done. A clear disinfection protocol was also outlined. Only the figures need to be in high quality for publication. Others are okay.

6. PLOS authors have the option to publish the peer review history of their article ([what does this mean?](https://journals.plos.org/plosone/s/editorial-and-peer-review-process#loc-peer-review-history)). If published, this will include your full peer review and any attached files.


If you choose “no”, your identity will remain anonymous but your review may still be made public.


**Do you want your identity to be public for this peer review?** For information about this choice, including consent withdrawal, please see our [Privacy Policy](https://www.plos.org/privacy-policy).

Reviewer #1: No

Reviewer #2: **Yes:** Mohd Ridha Muhamad

[NOTE: If reviewer comments were submitted as an attachment file, they will be attached to this email and accessible via the submission site. Please log into your account, locate the manuscript record, and check for the action link "View Attachments". If this link does not appear, there are no attachment files.]

While revising your submission, please upload your figure files to the Preflight Analysis and Conversion Engine (PACE) digital diagnostic tool, <https://pacev2.apexcovantage.com/>. PACE helps ensure that figures meet PLOS requirements. To use PACE, you must first register as a user. Registration is free. Then, login and navigate to the UPLOAD tab, where you will find detailed instructions on how to use the tool. If you encounter any issues or have any questions when using PACE, please email PLOS at <a href="mailto:figures@plos.org">figures@plos.org. Please note that Supporting Information files do not need this step.

**Response:**

Dear *PLOS ONE* editorial and Review Members,

PLOS ONE Decision: Revision required [PONE-D-24-08899] - [EMID:ab6d6df073dad6b3]

Thank you, for the suggestions to improve our manuscript – please find our responses to the reviewers’ suggestions, comments, and recommended areas of improvement below. We have highlighted in bold the reviewer’s comments point by point and our statements are provided below each recommendation.

We value these insights and would welcome review of these responses and to our updated manuscript, (**Development and Application of Decontamination Methods for the Re-Use of Laboratory Grade Plastic Pipette Tips**; PONE-D-24-08899).

Also, the funders had no role in study design, data collection and analysis, decision to publish, or preparation of the manuscript.

Again, thank you for your time and recommendations.

Sincerely,

Sang Hyuk Lee Ph. D

sleejhu361@gmail.com

**Reviewer #1:** General Comment.

The comparison between traditional methods of cleaning, like detergents, and more recent ones, like UV or CAP, is a subject of outmost interest and value in public health. For instance, CAP have been extensively researched in the last 20 years for different applications, but its actual implementation has evolved more slowly, precisely due to the requirement of more studies about its performance.
The paper has strong points like the organized structure of the manuscript, the thorough description of the protocol and elements used in the biological experiments, as well as the presentation of the results (graphs and tables).
My main concern with the manuscript is that I do not think the authors did a good job in comparing the performance of the three methods. There are parameters that are not consistent, like the number of samples, or seem to be chosen arbitrarily. In addition, there is the perception of lack of knowledge in the explanation of the results that were obtained. Although this is not a physics paper, the authors should have a minimum basic knowledge of the methods they are comparing.
Finally, the paper would benefit from a review by a native speaker who could improve the text and make it more natural and fluid.

We appreciate both the positive comments and constructive suggestions. We agree with the reviewer that the designs of our projects consisted of different periods of exposure and variable sample conditions or numbers. When feasible we implemented efforts to retain similar experimental conditions. However, and as example the differences in modalities required variable exposure periods or sample numbers.

Specifically, CAP was exposed for shorter intervals due to previous testing by our collaborators at periods of time that were comparable to those we tested. This is due to the specificities of this specific CAP modality that requires a shorter exposure time to prevent material damage while still providing efficacious surface treatment to plasticware.

We agree the detailed physics of these modalities are worth attention. Therefore, we have provided specific references to the CAP technology developed by our collaborator from Dr. Michael Keidar’ s lab that can also be found here:

Soni, V., et al., In Vitro and In Vivo Enhancement of Temozolomide Effect in Human Glioblastoma by Non-Invasive Application of Cold Atmospheric Plasma. Cancers (Basel), 2021. 13(17).

Soni, V., et al., Theranostic Potential of Adaptive Cold Atmospheric Plasma with Temozolomide to Checkmate Glioblastoma: An In Vitro Study. Cancers (Basel), 2022. 14.

However, this study focused on the development of streamlined methods as well as methods to quantify their efficacy, particularly (1) the elimination of genomic material that could influence downstream diagnostic assays and (2) the interference of decontamination modalities in the efficacy of plasticware (i.e. absence of residuals that could affect PCR based assays ). To do this, we measured for the log reduction and turnover ratio of microbial genetic material using RT-PCR to evidence appropriate decontamination.

We have reviewed the manuscript for appropriate language and editing considerations, thank you for this suggestion.


 Specific comments.

Line 45 and 48: “the highest turnover ratio (95.9 %) ... the second highest turnover ratio (98.4 %)”. There is an incoherence in that statement.

These lines are updated accordingly:

Detergent/washing methods showed the second highest turnover ratio (95.9 %) and log reduction (5.943)... Ozone vapor at 14400 PPM * minutes showed the highest turnover ratio (98.4 %) and log reduction (4.511).


Line 48: 14400 PPM * minutes, remove the ‘s’.

Updated.

Lines 50 to 52: “Relatively, lower turnover ratio and log reduction of CAP could be attributed to development/optimization of treatment conditions, including increases in exposure time and relative to tip positioning.” It is very difficult to make sense of this sentence, please rewrite.

These lines are updated accordingly:

Relatively, lower turnover ratio and log reduction of CAP could be improved by optimization, such as increasing the exposure time. Future testing would provide fine-tuned conditions for CAP-specific decontamination of plasticware. In this study we were able to provide fundamental insight into a non-traditional decontamination method for single-use plasticware that could render these products reusable.

Line 73: instead of using “[8]; Alconox® is a widely used laboratory cleaner” I would suggest using “[8], which is a widely used laboratory cleaner.” to make the reading more fluid.

Updated.

Lines 75 and 76: The authors wrote “ozone is a reactive allotrope of oxygen with a lone unpaired electron (free radical) that dimerize with valence electrons on other molecules.” That statement is not correct, please check.

Agreed and updated.

ozone is a triple oxygen molecule and act as a powerful oxidant.

Lines 84 and 85: The authors describe CAP as “a thermal non-equilibrium state between heavy positive ions and electrons achieved [10, 11] through rapid atmospheric pressure discharge.” The statement is not entirely accurate. The presence of negative ions in plasma is very important for Electronegative gases like oxygen and therefore, air. In the method presented in the manuscript, plasma is ignited first using helium gas. However, the decontamination effect is achieved through reactive species of oxygen and nitrogen formed through ionization of the surrounding air.

In addition, they should explain briefly and in a very simple (basic) form how the CAP machine works, or its principle of operation (DBD). In lines 243 and 244 they mention the voltage and frequency used, but without said explanation, that information is entirely useless for the reader.

I appreciate the attention to detail. Please note that the cold plasma device was provided from our collaborator who shared extensive documentation for their applications as described in these publications:

Soni, V., et al., In Vitro and In Vivo Enhancement of Temozolomide Effect in Human Glioblastoma by Non-Invasive Application of Cold Atmospheric Plasma. Cancers (Basel), 2021. 13(17).

Soni, V., et al., Theranostic Potential of Adaptive Cold Atmospheric Plasma with Temozolomide to Checkmate Glioblastoma: An In Vitro Study. Cancers (Basel), 2022. 14.

Since this study focuses on the use of streamlined protocols with confounding parameters defined, the level of information regarding CAP was intended to provide a high-level overview of the technology and its fundamental traits that would contribute to decontamination, safely, with plasticware.

Line 87: “CAP maintains temperatures of 25- 45°C…”. Please, add references.

Updated.

Lines 94 to 96: The paragraph should be rewritten, because it is not easy to understand.

These lines are updated accordingly:

This investigation compared the efficacies of genetic material removal from pipette tips commonly used for molecular assays, like polymerase chain reaction (PCR). The decontamination methods tested are a common laboratory detergent and steam sterilization, ozone vapor, and CAP. Commonly used laboratory and industrial applications were replicated [11, 12, 14, 15] to compare decontamination modalities.

Lines 245 to 247: The authors mentioned that the pipette tips were placed 2 to 5 cm under the plasma jet for 1 minute. How and why such distance and exposure time were selected.

The distance of the jet and pipette tip accounts for fluctuating flame distance due to minor fluctuation in helium flow rate—prior to experimentation we measured for the appropriate distance from the plume (flame) from CAP and the tip. Additionally, we had a single jet CAP exposure apparatus, a manifold or similar apparatus could have optimized and streamlined exposure and minimized human error. These designs were in production at the time of this study and will be discussed in future publications and research efforts.

Line 368 (table 4): Why are there inconsistencies in the number of samples used (73 vs 63)? The turnover ratio is better for O3 14400 ppm than for detergent, but the authors claim otherwise.
I do not think the authors are making a fair comparison or assessment of the cleaning methods proposed, since they seem to have chosen arbitrary exposure distance and time for CAP. They soaked the tip racks for 30 minutes in the detergent solution and used the same time exposure for O3 14400 ppm; however, for CAP they exposed the pipettes for 1 minute, without further explanation to justify that decision. Furthermore, as mentioned before, CAP decontamination is mostly achieved through the action of ROS/RNS (O3 being among them). UV radiation may have some effect if the object is placed near enough to the plasma luminescence. It would have been very useful if the authors could have roughly measured the concentration of at least some of the ROS/RNS, although I understand that this is only possible if they have access to some specialized equipment. However, the authors at least should have done a preliminary study to establish a minimum exposure time, after which the variations in log reduction are not considerable.

The variation in sample numbers was mainly to maintain experimental ethics, in which case we did not want to cherry pick data we collected for the cohort that we tested with Alconox detergent. Otherwise, we performed a power test to identify the appropriate 63 for this study with the assistance of Dr. Xiao Fu (the statistician who assisted with the appropriate samples numbers to test for significance).

The exposure duration of CAP was an educated decision following preliminary test results and discussions between our collaborators (the developers of the CAP technology) and our research team. We value the reviewer’s insight into the concentration of ROS/ANS produced by CAP—the concentrations of both were previously evaluated by our collaborators and this information was shared with our team and considered in the design of these experiments. Specifically, the graph below shows the concentration of ROS/ANS—these data are currently in-submission for a separate manuscript. Additionally, as the end-goal for this study was to examine the removal of genomic material from these plasticware, the efficacy of this specific examination was central to our work.

Figure Description: OES analysis of the 5×1 array multiplasma jet device. (A) Close to nozzle - the spectrum reveals the presence of RONS generated by the plasma jets, with specific peaks corresponding to different species such as oxygen (O), hydroxyl radicals (OH), nitric oxide (NO), and nitrogen (N2). (B) Middle of plasma, (C) Measurement of OES at plasma tip. (D) No plasma contact. The intensity and distribution of these peaks provide insights into the efficiency and uniformity of reactive species production.

**Reviewer #2:** The experiment and data presented is good to be accepted as it is. The experiment and statistical analysis were done. A clear disinfection protocol was also outlined. Only the figures need to be in high quality for publication. Others are okay.

I appreciate the positive comments. Better images are provided for the revision submission.
